# The Effect of Sleep Restriction, With or Without Exercise, on Skeletal Muscle Transcriptomic Profiles in Healthy Young Males

**DOI:** 10.3389/fendo.2022.863224

**Published:** 2022-07-22

**Authors:** Wentao Lin, Nicholas J. Saner, Xiquan Weng, Nikeisha J. Caruana, Javier Botella, Jujiao Kuang, Matthew J-C. Lee, Nicholas A. Jamnick, Nathan W. Pitchford, Andrew Garnham, Jonathan D. Bartlett, Hao Chen, David J. Bishop

**Affiliations:** ^1^ College of Exercise and Health, Guangzhou Sport University, Guangzhou, China; ^2^ Institute for Health and Sport, Victoria University, Melbourne, VIC, Australia; ^3^ Human Integrative Physiology, Baker Heart and Diabetes Institute, Melbourne, VIC, Australia; ^4^ Department of Biochemistry and Pharmacology and Bio21 Molecular Science and Biotechnology Institute, The University of Melbourne, Parkville, VIC, Australia; ^5^ Metabolic Research Unit, Institute for Mental and Physical Health and Clinical Translation, School of Medicine, Deakin University, Geelong, VIC, Australia; ^6^ School of Health Sciences, University of Tasmania, Launceston, TAS, Australia

**Keywords:** high-intensity interval exercise (HIE), transcriptomics, sleep loss, skeletal muscle, mitochondria, circadian rhythm, sleep restriction, inflammation

## Abstract

**Background:**

Inadequate sleep is associated with many detrimental health effects, including increased risk of developing insulin resistance and type 2 diabetes. These effects have been associated with changes to the skeletal muscle transcriptome, although this has not been characterised in response to a period of sleep restriction. Exercise induces a beneficial transcriptional response within skeletal muscle that may counteract some of the negative effects associated with sleep restriction. We hypothesised that sleep restriction would down-regulate transcriptional pathways associated with glucose metabolism, but that performing exercise would mitigate these effects.

**Methods:**

20 healthy young males were allocated to one of three experimental groups: a Normal Sleep (NS) group (8 h time in bed per night (TIB), for five nights (11 pm – 7 am)), a Sleep Restriction (SR) group (4 h TIB, for five nights (3 am – 7 am)), and a Sleep Restriction and Exercise group (SR+EX) (4 h TIB, for five nights (3 am – 7 am) and three high-intensity interval exercise (HIIE) sessions (performed at 10 am)). RNA sequencing was performed on muscle samples collected pre- and post-intervention. Our data was then compared to skeletal muscle transcriptomic data previously reported following sleep deprivation (24 h without sleep).

**Results:**

Gene set enrichment analysis (GSEA) indicated there was an increased enrichment of inflammatory and immune response related pathways in the SR group post-intervention. However, in the SR+EX group the direction of enrichment in these same pathways occurred in the opposite directions. Despite this, there were no significant changes at the individual gene level from pre- to post-intervention. A set of genes previously shown to be decreased with sleep deprivation was also decreased in the SR group, but increased in the SR+EX group.

**Conclusion:**

The alterations to inflammatory and immune related pathways in skeletal muscle, following five nights of sleep restriction, provide insight regarding the transcriptional changes that underpin the detrimental effects associated with sleep loss. Performing three sessions of HIIE during sleep restriction attenuated some of these transcriptional changes. Overall, the transcriptional alterations observed with a moderate period of sleep restriction were less evident than previously reported changes following a period of sleep deprivation.

## Introduction

The increased time demands imposed on modern society mean that many adults do not meet nightly sleep recommendations of 7 to 9 h per night (1, 2). Despite the importance of sleep for the maintenance of good health (3), the effects of inadequate sleep are still not completely understood. Nonetheless, the consequences of not obtaining the recommended amounts of sleep on aspects of metabolic health are becoming increasingly apparent. Several epidemiological studies have demonstrated that short sleep duration is associated with a greater risk of developing diabetes, cardiovascular disease, and dementia, as well as an increase in all-cause mortality (4–6).

These epidemiological findings are now supported with evidence from laboratory-controlled studies that have investigated the detrimental physiological effects associated with either one night of sleep deprivation (24 h of sustained wakefulness) or extended periods of recurrent sleep restriction (reductions of time in bed (TIB) per night) (7–15). These studies indicate that sleep loss can have detrimental effects within peripheral tissues, such as skeletal muscle (12, 16–19). Indeed, we have previously reported changes in skeletal muscle mitochondrial function, protein synthesis, the expression of core clock gene expression (that regulate circadian rhythms), and glucose metabolism following five nights of 4 h sleep in young males (14, 15), which are supported by findings from many others too (7, 11, 20, 21). Despite these findings, the skeletal muscle transcriptomic responses that may contribute to these effects require further elucidation.

Transcriptomic approaches allow for the assessment of genome-wide changes in gene expression, with the potential to offer insight into the early transcriptional changes that may regulate the functional consequences of sleep loss. A seminal study by Cedernaes etal. (13) reported changes in the skeletal muscle transcriptome following 24 h of sleep deprivation. Following the intervention, 118 mRNA transcripts were found to have altered expression levels; these were primarily related to increases in inflammatory pathways, decreases in genes associated with oxidative phosphorylation, alterations to core circadian clock genes, and a transcriptional signature suggestive of reductions in protein synthesis pathways. While sleep deprivation can occur, a more common problem is repeated nights of sleep restriction (e.g., 4 h of sleep per night). Further research is required to investigate whether the changes reported by Cedernaes et al. (13) are confined to severe sleep deprivation or also occur in response to less extreme models of sleep restriction.

The identification of the many detrimental responses to the stress of insufficient sleep highlights the need to investigate interventions that are capable of mitigating these effects (18). In fact, bouts of recovery sleep are not always sufficient to mitigate the detrimental effects of sleep loss on metabolic health (22–25). However, exercise improves glucose tolerance (26, 27) and mitochondrial function (28–32), and can alter circadian rhythms (33, 34). Data from our lab (utilising the same design as the present study) has also demonstrated that high-intensity interval (HIIE) exercise is able to mitigate the detrimental effects of sleep restriction (5 nights, 4 h TIB each night) on glucose tolerance, mitochondrial function, circadian rhythms, and both sarcoplasmic and myofibrillar protein synthesis (14, 15, 18). Further studies have also demonstrated a protective effect of HIIE on glucose tolerance if performed in the weeks prior to sleep deprivation (35), or immediately following sleep restriction (36). These exercise-induced adaptations have been attributed to large-scale alterations to the skeletal muscle transcriptome (including oxidative phosphorylation, inflammatory, protein synthesis, and glucose metabolism signalling pathways), which occur in the hours following exercise (29, 37–39). However, the transcriptional response to a period of insufficient sleep combined with exercise has never been investigated.

Accordingly, the overall aim of this study was to investigate the transcriptomic changes that occur in human skeletal muscle following sleep restriction (5 nights, 4 h TIB per night), with or without HIIE. We hypothesised that sleep restriction would down-regulate transcriptional pathways associated with glucose metabolism and mitochondrial function, but that performing exercise would mitigate these effects. This information will improve our understanding of the underlying mechanisms that regulate sleep-loss-induced changes in metabolic health and help to elucidate the mechanisms by which exercise may ameliorate these changes.

## Methods

### Ethics Approval

All procedures involved in this study conform to the standards set by the latest revision of the Declaration of Helsinki (except for registration in a database) and were approved by the Victoria University Human Research Ethics Committee (HRE15-294).

### Participants

Twenty healthy, recreationally active males, aged between 18 and 40 years of age, volunteered to participate. Eligible participants 1) were not taking any medications, 2) were not performing shift work (within the previous three months), 3) had regular sleeping habits (6 to 9 hours per night) and no previously diagnosed sleep disorders, 4) had not travelled overseas in the previous two months, and 5) had a body mass index between 19 and 30 (kg/m^2^).

### Study Overview

Eligible participants attended the exercise physiology laboratory for baseline anthropometric measurements (i.e., height and body mass), and aerobic fitness testing (peak oxygen uptake $\left[\dot{V}{\text{O}}_{2\text{peak}}\right]$ and peak power output [Ŵ_Peak_]) that was performed to volitional exhaustion on an electronically braked cycle ergometer (Excalibur, V2.0; Lode, Groningen, Netherlands), using an incremental ramp protocol (30 W/minute). Following baseline testing, participants were allocated *via* minimisation into one of the three experimental groups, in a counterbalanced order, so as to minimise differences in between-group baseline measures for age, body mass index (BMI), habitual sleep duration, and $\dot{V}{\text{O}}_{2\text{peak}}$ (**Table 1**).

**Table 1:** Baseline characteristics of participants.

	Group characteristics			
	NS (n = 6)	SR (n = 7)	SR+EX (n = 7)	*P* Value
**Age** (y)	24.2 ± 3.9	25.1 ± 4.8	24.3 ± 3.5	*P* = 0.895
**Height** (m)	1.78 ± 0.09	1.79 ± 0.06	1.82 ± 0.05	*P = 0.638*
**BMI** (kg/m^2^)	24.3 ± 3.1	22.6 ± 2.4	25.2 ± 2.2	*P* = 0.179
$\dot{V}O_{2\mathrm{peak}}$ **(mL.kg^-1^.min^-1^)**	44.9 ± 10.9	48.4 ± 6.3	48.7 ± 4.9	*P* = 0.651
$\dot{V}O_{2\mathrm{peak}}$ **(mL.min^-1^)**	3422 ± 597.5	3681 ± 17.3	4037 ± 377.2	*P* = 0.076
**Fasting plasma glucose** (mmol.L^-1^)	4.9 ± 0.3	5.1 ± 0.3	5.2 ± 0.2	*P* = 0.423
**Day 3 - Glucose AUC** (A.U)	653 ± 185	659 ± 80	632 ± 50	*P* = 0.889
**Day 8 - Glucose AUC** (A.U)	594 ± 129	835 ± 54*	696 ± 72^#^	*P* < 0.001
**Day 3 – HOMA-IR**	1.5 ± 0.3	1.9 ± 0.5	1.4 ± 0.7	*P* = 0.164
**Day 8 – HOMA-IR**	1.7 ± 0.5	1.9 ± 0.8	1.4 ± 0.6	*P* = 0.414
**Habitual sleep duration** (h:min/night)	7:38 ± 0:50	7:15 ± 0:41	7:12 ± 0:41	*P* = 0.884
**MEQ score**	46 ± 7	46 ± 8	59 ± 7	*P* = 0.095
**Daily step count** (Habitual)	12827 ± 4653	11569 ± 1854	11831 ± 919	*P* = 0.822
**Daily step count** (Study)	11043 ± 2553	10139 ± 2035	11344 ± 2357	*P* = 0.702

Values are mean ± sd. There were no significant differences between the three groups for any of the baseline characteristics. Data pertaining to VO_2peak_, glucose AUC, HOMA-IR, and step counts have been reported previously and have been reanalysed for this study. NS – Normal sleep, SR – Sleep restriction, SR+EX – Sleep Restriction and Exercise, BMI – body mass index, AUC – area under the curve, HOMA-IR – Homeostatic model assessment of insulin resistance index, MEQ – morningness-eveningness chronotype question. * denotes significantly different from NS group, ^#^ denotes significantly different from SR group, P < 0.05.

**Figure 1:**
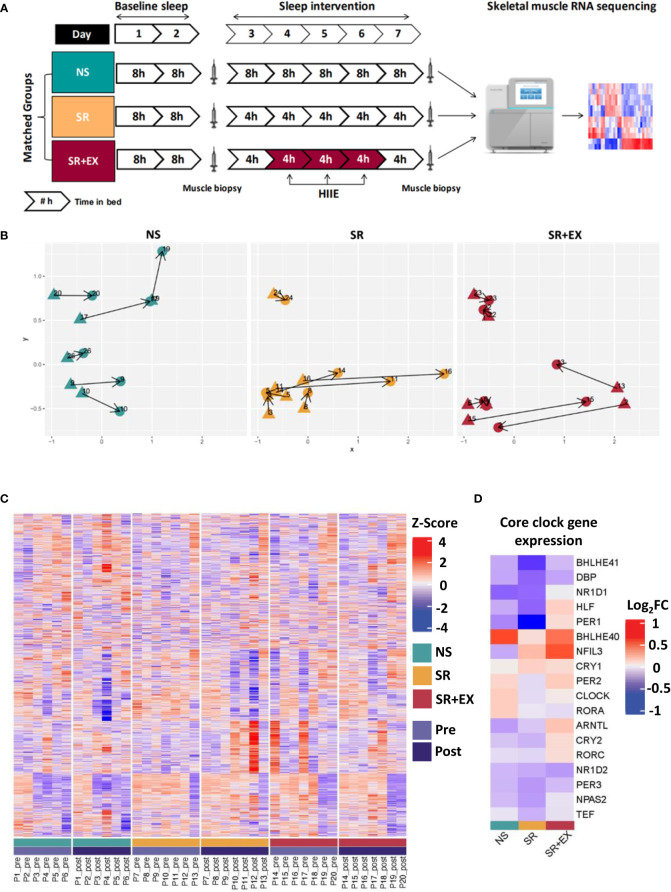
**(A)** Study protocol and analysis overview. Participants were matched into one of three experimental groups. The study consisted of two nights of baseline sleep (8 h TIB), prior to 5 nights of either 8 h TIB (NS), or 4 h TIB (SR and SR+EX). The SR+EX group performed 3 sessions of HIIE on Day 4, 5 and 6. Skeletal muscle biopsies were collected pre- and post-intervention and underwent transcriptomic analysis. **(B)** Multidimensional scaling (MDS) plot showing distances between each group (blue = NS, yellow = SR, red = SR+EX), with each biopsy time points (pre- (triangles) or post-intervention (circles)). **(C)** Heatmap of relative expression levels of transcripts identified within the study. Individuals were grouped according to their intervention and time point (pre or post). **(D)** Changes in expression of core circadian clock genes for each group (Log_2_FC from pre- to post-intervention). Groupings include green = normal sleep [NS], yellow = sleep restriction [SR], red = sleep restriction and exercise [SR+EX], with Pre [light purple] and Post [dark purple]. NS – Normal Sleep, SR – Sleep Restriction, SR+EX – Sleep Restriction and Exercise, HIIE – High-intensity interval exercise, TIB – Time in bed.

The study consisted of an eight-night stay within a temperature-controlled (22°C) sleep laboratory. All groups completed two initial nights of baseline sleep (8 h TIB from 23:00 h to 07:00 h), followed by a five-night intervention period, during which the Normal Sleep (NS) group spent 8 h TIB (23:00 h to 07:00 h), while both of the Sleep Restriction (SR) and Sleep Restriction and Exercise (SR+EX) groups spent 4 h TIB per night (03:00 h to 07:00 h). Between 23:00 h and 03:00 h, lighting was dimmed to below 15 lux to reduce the effect of lighting on circadian rhythms (40). The SR+EX group also performed three exercise sessions during the intervention period on days 4, 5, and 6 at 10:00 h. Following the intervention period and completion of testing, all participants remained at the sleep facility and completed a final night of *ad libitum* recovery sleep. Participants were monitored throughout the protocol and provided with a standardised diet consisting of fixed proportions (relative to body mass) of carbohydrates (4.5 g.kg^-1.^d^-1^), protein (1.5 g.kg^-1.^d^-1^) and fat (1 g.kg^-1.^d^-1^). All mealtimes were kept constant each day (breakfast - 08:00 h, snack - 10:30 h, lunch - 12:30 h, snack – 16:30 h, dinner – 19:00 h, and snack – 21:30 h). An overview of the study protocol is shown in **Figure 1A**.

### Sleep, Chronotype, and Physical Activity Monitoring

Sleep was assessed for one week prior to and then throughout the study using wrist-watch actigraphy devices (Actiwatch 2, Philips Respironics, Murrysville, PA, USA) (41). The habitual sleep monitoring occurred within two weeks of the study commencing and participants were instructed to maintain habitual sleep habits in the week preceding the study. Prior to the study, participants completed the Horne-Osteberg morningness-eveningness questionnaire to assess chronotype (42). All participants were classified as having either a moderate evening (n=2), intermediate (n=14), or moderate morning (n=4) chronotype. Habitual daily step counts were monitored using validated step-counting applications on the participants’ personal mobile phone devices (i-Health app, Apple Inc., Cupertino, CA, USA; and Samsung Health, Samsung Electronics Co., Ltd., Suwon, South Korea) and these step counts were replicated throughout the study using the same devices (data reanalysed from (14). During the study, participants were told of their daily step count requirements and prompted to meet these targets each day (**Table 1**).

### Statistical Analysis

A 2-way repeated measures ANOVA (group x time) was used to assess differences in mean nightly sleep duration for the baseline period and sleep intervention period. Differences for between group participant characteristics were assessed with a one-way ANOVA. Where significant differences were observed Tukey, *post-hoc* analysis was conducted to investigate differences between groups. *P* values < 0.05 indicate statistical significance. Statistical analysis of these parameters was conducted using the statistical software package Graphpad Prism (V7.03).

### High-Intensity Interval Exercise

The HIIE protocol consisted of 10 × 60-second intervals performed on a cycle ergometer (Velotron, Racer-Mate, Seattle, WA, USA) at 90% of each participant’s Ŵ_peak_ (at ~70 RPM). Each interval was interspersed with 75 seconds of active recovery at 60 W. Each session started with a 3-minute warm up at 60 W. The mean power per interval was 334 ± 29 W and the mean HR throughout the protocol was 157 ± 12 bpm.

### Muscle Biopsies

On Day 3 and Day 8 of the study, muscle biopsies were sampled from the *vastus lateralis* muscle using a suction-modified Bergström needle, and under local anaesthesia of the skin and fascia (1% lidocaine). All samples were collected at 10:00 h (three hours after waking up). The post-intervention sample was collected 48-h after the final HIIE session from the participants in the SR+EX group. All samples were collected following the completion of a fasted oral glucose tolerance test (75 g glucose) (**Table 1**). All samples were immediately frozen in liquid nitrogen and stored at -80°C.

### RNA Extraction

Frozen muscle samples (10 to 20 mg) were removed from -80°C storage and placed on dry ice. RNA extraction was performed using RNeasy Plus Universal Mini Kit (Qiagen, Valencia, USA) as previously described (43). In order to increase RNA yield, kit instructions were modified by replacing ethanol with 2-propanol to precipitate the RNA. A genomic DNA elimination step was included in the kit to remove genomic DNA from the total RNA. Prior to storage, separate aliquots were taken for RNA quantification and RNA integrity testing.

### RNA Quantification

RNA samples were quantified using a Nanodrop spectrophotometer (Thermo Fisher Scientific). 1 μL of each RNA sample was placed onto the spectrophotometer and readings for RNA concentration (ng/μL), the A260/280 ratio and A260/230 ratio was recorded. No further dilution of RNA samples was used.

### RNA Integrity

Assessment of RNA integrity was performed using an automated microcapillary electrophoresis system (Experion, Bio-Rad Laboratories, Hercules, CA). This protocol was performed as per the manufacturer’s instructions and generated an RNA quality Indicator score (RQI) from 1 to 10. Any sample with an RQI greater than 7 was considered to be of good integrity and the RNA intact (43).

### RNA Sequencing

The library construction and RNA sequencing was performed by Beijing Genomics Institute (Shenzhen, China) as described previously (44). In total, approximately 1 μg total RNA was used for library construction. For library preparation, the poly(A) + mRNA molecules were purified using oligo (dT) magnetic beads. The mRNA was fragmented into small pieces and the double-strand cDNA was synthesized using N6 random primers. These cDNA fragments were added with a single ‘A’ base and subsequently ligated to the adapter. The ligation product was purified and enriched with PCR amplification to yield the final cDNA library. The cDNA library was sequenced using BGISEQ-500 system with a paired-end sequencing length of 100 bp. Raw RNAseq counts for each sample are available in **Supplementary Table 1**.

### Assembly and Bioinformatic Analysis of RNA-Seq Data:

Cleaned sequence reads were aligned against the *Homo sapiens* genome (Build version HG38). *Salmon* (1.4.0) was used to map reads to the genomic sequences. Counts of read mapping to each known gene were summarised to provide the matrix used for further analysis. Genes lowly expressed were discarded from downstream analysis resulting in 14,917 genes across samples. Count data was normalised using calcNormFactors with trimmed value of means (TMM) in the *edgeR* package in R. For downstream RNA-seq analysis the R package *limma* (45) was used to conduct the differential expression analysis from count data. Differential expression analysis was attempted between the pre- and post-intervention biopsy for each group (**Supplementary Table 2**). The transcriptomic data has been deposited in the NCBI and can be found under the BioProject PRJNA807098.

Differential expression analysis was performed between each subsequent biopsy to show comparative changes between pre- and post-intervention for each group, though all comparisons were accounted for within the analysis. The resulting differential expression values were filtered for an adjusted *P* value < 0.05 using the Benjamini-Hochberg method. Heatmaps were visualised using hierarchical clustering for the rows using the “ward.D” method and supervised grouping for the columns into each of the three conditions.

### Gene Set Enrichment Analysis:

**Figure 2:**
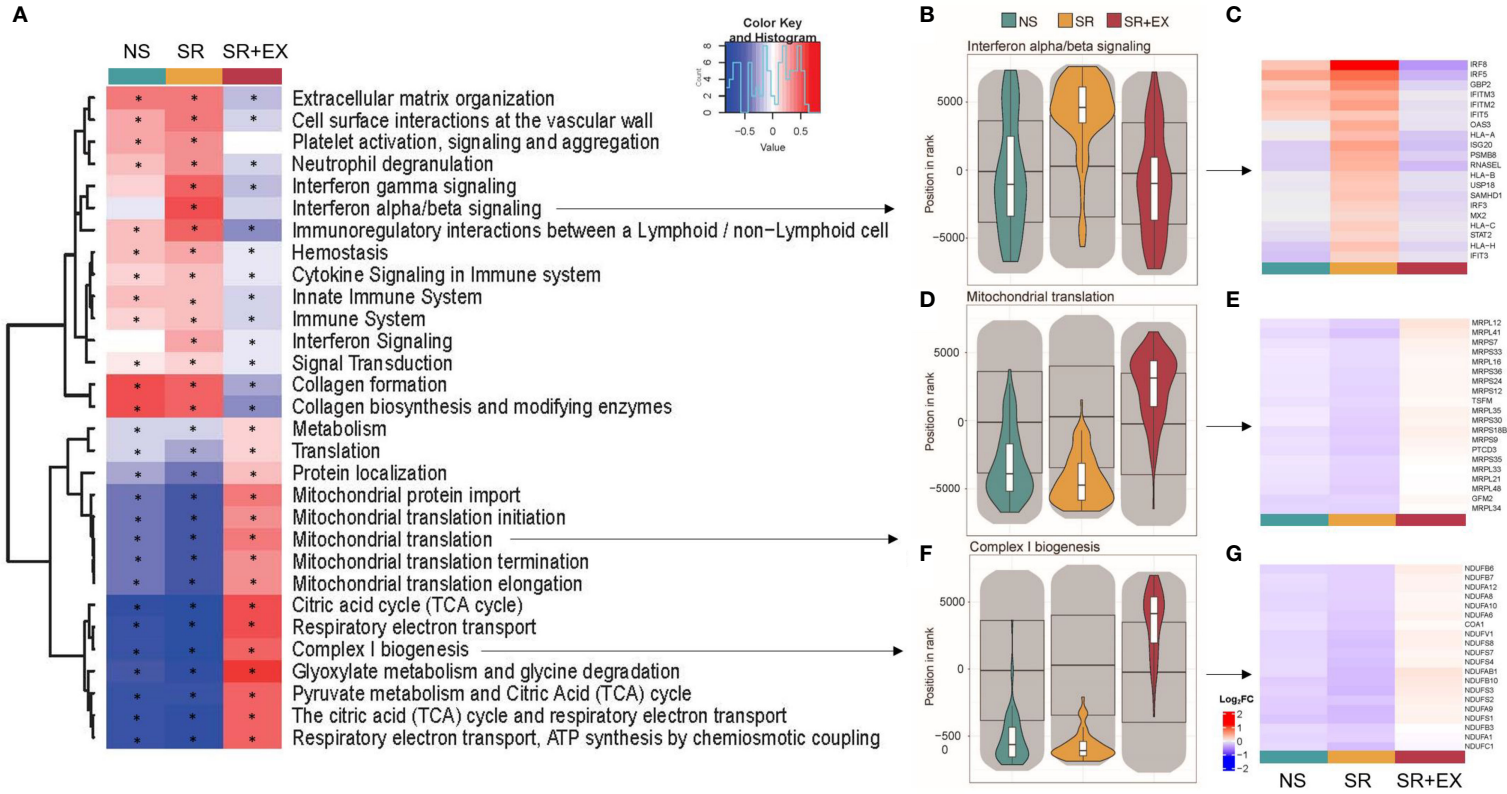
Gene set enrichment analysis (GSEA). **(A)** Heat map representing the top 30 significantly altered gene sets from the ‘REACTOME’ gene ontology (based on MANOVA analysis using the *mitch* R package (46). **(B, D, F)** Violin plots representing the direction of expression in each group for b) Interferon alpha/beta signalling, **(D)** Mitochondrial translation, and **(F)** Complex I biogenesis. **(C, E, G)** heat maps for the 20 leading-edge genes for the corresponding violin plots (Log_2_FC from pre- to post-intervention). Normal Sleep (NS), Sleep Restriction (SR), and Sleep Restriction and Exercise (SR+EX). * Denotes significant within group difference from pre-intervention, adjusted *P*<0.05.

To gain further insight into the different effects of the intervention we performed a multi-contrast gene set enrichment analysis (GSEA) using the R package *mitch (v.1.4.0) (* 46 *)*. This package allows the detection of gene set enrichments that show similar or divergent responses across contrasts (groups). The input data provided was the log_2_FC obtained from pre- to post-intervention in each group using the normalised RNA-seq data from *edgeR*. Multi-contrast analysis was performed using the statistical test of Multiple Analysis of Variance (MANOVA) incorporated in the *mitch* package. The top 30 pathways based on this MANOVA analysis are displayed in **Figure 2**. The analysis was performed on the REACTOME gene sets (version 2021-06-08) and an adjusted *P* value < 0.05 was deemed significant. Enrichment score (ES) was also obtained and ranged from -1 to 1. REACTOME pathway analysis and GSEA data is available in **Supplementary Table 3**.

To further compare our data set to previously reported changes in skeletal muscle transcripts following 24 h of sleep deprivation (SD) (13), we created two gene sets from that study’s differentially expressed genes. Their down-regulated gene set comprised of 98 genes and their up-regulated gene set contained 20 genes (**Supplementary Table 4**), and we named them as ‘SD – Down’ and ‘SD – Up’, respectively. We performed GSEA, on the newly created gene sets, using pre-ranked genes based on their Pre to Post log_2_FC using the *fgsea* (v.1.18.0) R package (47). Pre-ranked samples were permuted 1 million times for GSEA, and a Benjamini-Hochberg adjusted *P* < 0.05 was considered significant. The participant cohort for the Cedernaes etal. (13) study was very similar to the present study and included a young healthy male cohort (n = 15, age; 22.3 ± 0.5 years, BMI; 22.6 ± 0.5 kg/m^2^), with regular self-reported sleep habits (7 – 9 hours per night), no extreme morning/evening chronotypes, and no self-reported chronic medical conditions. The sleep condition in the study was also similar to the present study and consisted of an 8.5 h sleep opportunity and the average sleep duration on this night was 8.0 h ± 3 min. As in the present study, the muscle biopsies were collected during the morning (between 8 am and 10 am).

### Results:

The total sleep time (TST) of all participants was assessed using wristwatch sleep actigraphy. There were no differences between groups for mean nightly TST during the baseline sleep period (TST ± sd, NS; 446 ± 26 min, SR; 450 ± 17 min, SR+EX; 457 ± 8 min, *P*>0.05). There was a significant interaction effect for TST (*P*<0.001) when comparing the baseline period to the sleep intervention period. During the intervention phase of the study, there was a significant difference between groups for nightly TST (NS; 450 ± 26 min, SR; 230 ± 6 min, and SR+EX; 234 ± 3 min, *P*<0.001). Compared to the NS group, there was a significant decrease in TST in the SR group (mean difference ± sd, [95% CI], *P* value, 220 ± 11 min, [197, 244 min], *P*<0.001) and SR+EX group (216 ± 11 min, [193, 239 min], *P*<0.001) during the intervention phase. There was no significant difference in TST between the SR and SR+EX groups (-4 ± 3 min, [-27, 18 min], *P*=0.950).

The changes in glucose tolerance (**Table 1**), myofibrillar protein synthesis, and skeletal muscle mitochondrial function reported in this cohort previously (14, 15), may be underpinned by changes within the skeletal muscle transcriptome. Therefore, to assess potential changes in gene expression in skeletal muscle that occur in response to a period of sleep restriction, with or without exercise, we performed transcriptomic RNA sequencing (RNAseq) on muscle samples collected pre- and post-intervention. In total, there were 14,917 genes detected with our analysis. Plotting each of the three groups within a multidimensional scaling (MDS) plot failed to separate out any discernible groups (**Figure 1B**). These results were further enforced by a lack of differentially expressed individual genes (adjusted *P* values < 0.05; Benjamini-Hochberg method) for any of the groups after the intervention had taken place (**Figure 1C**). Indeed, there were no significant changes in key circadian clock genes either (**Figure 1D**).

We then tested for gene sets of functional and biological importance that might be systematically altered in a coordinated manner in the skeletal muscle following our intervention using GSEA (as previously described; (48, 49). Similar approaches have been used where no discernible changes are observed at an individual gene level, but increased insight regarding alterations of underlying biological pathways can be obtained when changes across predefined gene sets are aggregated, (50, 51). We first used a multi-contrast analysis comparing the transcriptomic changes across groups using the REACTOME gene ontology (**Figure 2A**). This unbiased analysis revealed that pathways significantly enriched, and with the greatest enrichment score, were related to processes associated with the regulation of mitochondrial function and inflammation (**Figure 2B–G**). Among the pathways significantly upregulated in the SR group, were multiple inflammatory and immune system pathways (**Figure 2A**), including the interferon alpha/beta signalling (R-HSA-909733) pathway (IFNα/β) (enrichment score (ES), adjusted *P* value, IFNα/β ES=0.509, *P*<0.001), interferon gamma signaling (R-HSA-877300) pathway (IFNƴ) (ES=0.480, P<0.001), and the interferon signaling (R-HSA-913531) pathway (IFN) (ES=0.281, *P <*0.001). These same pathways were unchanged in the NS group (IFNα/β; ES=-0.042, *P*=0.598, IFNƴ; ES=0.112, *P* =0.093, and IFN; ES=0.002, *P* =0.951). However, in the SR+EX group there was no change in IFNα/β (ES=-0.109, *P*=0.175), but the IFNƴ (ES=-0.177, *P* =0.007) and IFN (ES=-0.100, *P*=0.026) pathways were negatively enriched. There was a significant up-regulation of mitochondrial-related pathways within the SR+EX group, including the tricarboxylic acid (TCA) cycle (R-HSA-71403) (TCA ES =0.576, *P*<0.001), Mitochondrial translation (MT) (R-HSA-5368287) (ES=0.373, *P*=<0.001), and Complex I biogenesis (CI) (R-HAS-6799198) (CI; ES=0.507, *P*<0.001). The tricarboxylic acid (TCA) cycle, Mitochondrial translation, and Complex 1 biogenesis gene sets were significantly down-regulated in both the SR (TCA; ES=-0.848, *P*<0.001, MT; ES=-0.625, *P*<0.001, and CI; ES=-0.812, *P*<0.001) and NS (TCA; ES=-0.-0.771, *P*<0.001, MT; ES=-0.419, *P*<0.001, and CI; ES=-0.676, *P*<0.001) groups. There was also a significant decrease in the Translation (R-HSA-72766) pathway in the SR (ES=-0.283, *P*<0.001) and the NS group (ES=-0.124, *P*<0.001), but there was an increase in this pathway in the SR+EX group (ES=0.114, *P*=0.001). In the top 30 significantly altered gene sets (**Figure 2A**), the enrichment score was larger in the SR group compared to the NS group in 28 of the pathways (other than the collagen formation (R-HSA-1474290) and collagen biosynthesis and modifying enzymes (R-HSA-1650814) pathways) (**Supplementary Table 3**); however, many pathways in the NS group were also significantly enriched in the same direction as the SR group.

**Figure 3:**
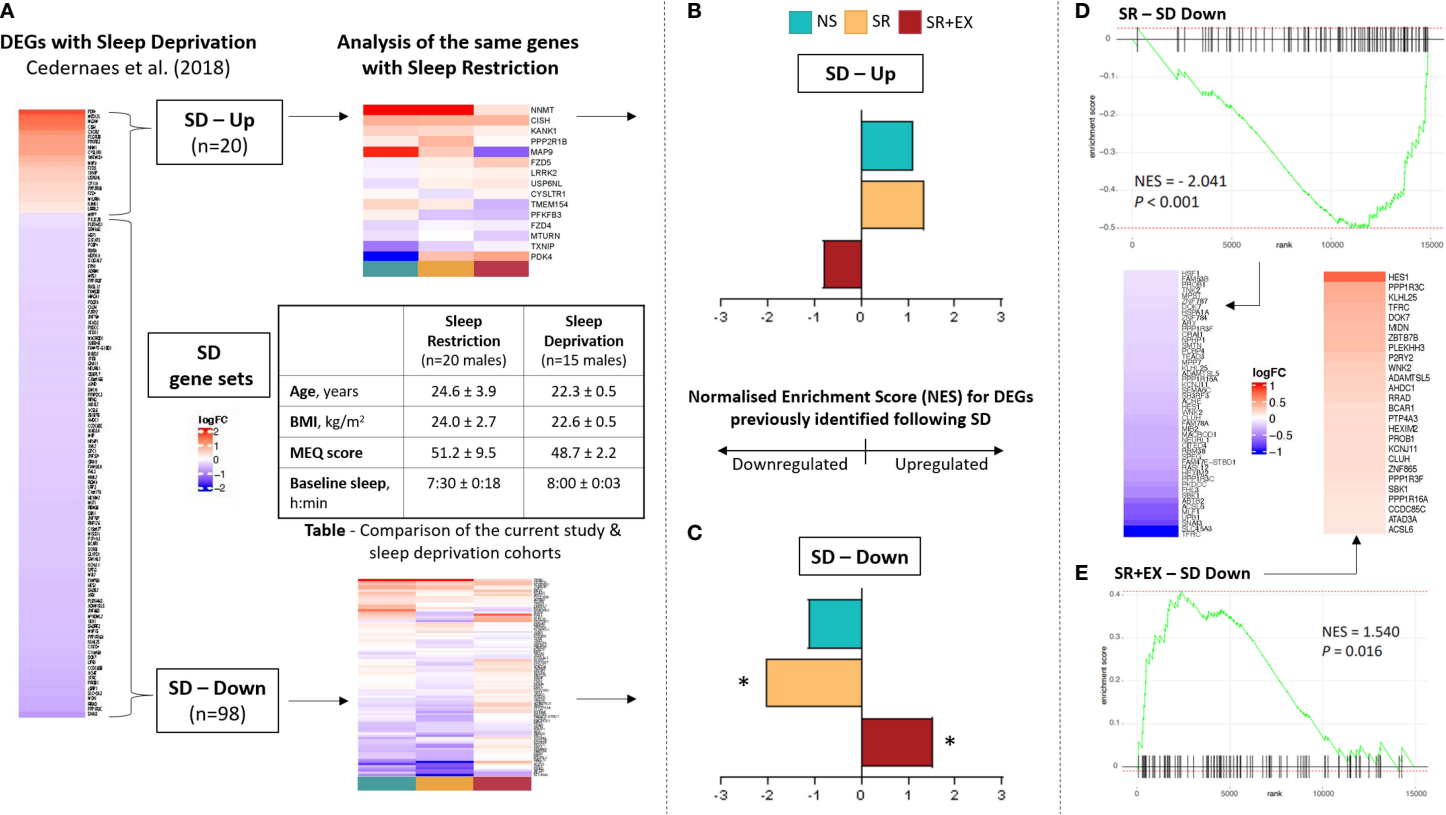
Comparison of our data set with previously reported transcriptional changes following 24 hours of sleep deprivation (13). **(A)** Overview of analysis workflow: two gene sets were created based on 1) Up-regulated and 2) Down-regulated genes in skeletal muscle following 24 h of sleep deprivation (SD) (created gene sets labelled as SD – Up and SD – Down, respectively). The enrichment of genes comprising SD-Up and SD-Down were then assessed in the current study cohort. Normalised enrichment score (NES) of our data set compared to the **(B)** SD – Up and **(C)** SD - Down gene sets. Rank and enrichment score of the SD - Down gene set in the **(D)** SR group and **(E)** SR+EX group, as well as heatmaps of leading-edge genes. Normal Sleep (NS), Sleep Restriction (SR), Sleep Restriction and Exercise (SR+EX), morningness-eveningness questionnaire score (MEQ score), differentially expressed genes (DEGs). *Denotes significant enrichment (adjusted *P* value <0.05).

Our multi-contrast analysis demonstrated that similar pathways were altered in the SR and SR+EX groups (**Supplementary Table 3**) compared to the previously reported changes in skeletal muscle transcripts with total sleep deprivation, which included the upregulation of KEGG pathways such as leukocyte transendothelial migration (hsa04670) and the downregulation of pathways such as oxidative phosphorylation (hsa00190) and ribosomes (hsa03010) (13). However, while the direction of enrichment in these pathways was similar in the SR group compared to the total sleep deprivation condition, the SR+EX group was enriched in the opposite direction. Next, we probed our data set to compare the enrichment, magnitude, and direction of change using the gene sets that were significantly up- and down-regulated following 24 h of sleep deprivation (referred to as, gene sets SD – Up and SD – Down) (**Figure 3**). In the SR group, there was a significant negative enrichment in the SD - Down gene set (Normalised Enrichment Score (NES), adjusted *P* value, NES=-2.041 *P*<0.001). However, there was no significant change in the SD - Up gene set in the SR group (NES=1.370, *P*=0.106). In the SR+EX group, there was a significant enrichment in the SD - Down gene set, but in the opposite direction (i.e., an increase) (NES=1.540, *P*=0.016). There was no significant change in the SR+EX group in the SD - Up gene set (NES=-0.818, *P*=0.706). There were no significant changes in either gene set in the NS group (Down; NES=-1.136, *P*=0.321, Up; NES=1.125, *P*=0.321).

## Discussion

We assessed changes in the skeletal muscle transcriptome in response to a period of sleep restriction (five nights, 4 h TIB per night), with or without HIIE in healthy males. We did not detect any significant changes in individual genes from pre- to post-intervention, which contrasts with previous reports of skeletal muscle transcriptional changes in response to one night of total sleep deprivation (13). These results suggest a period of moderate sleep restriction may not cause the same magnitude of changes to the skeletal muscle transcriptome as 24 h of total sleep deprivation. However, utilising GSEA, we provide evidence of small but significant changes in important transcriptional pathways. These pathways were specifically related to the regulation of interferon signalling and inflammatory/immune responses pathways following SR. However, these changes were absent or occurred in the opposite direction in the SR+EX group.

The transcriptional responses of skeletal muscle, a key metabolic organ, to a period of moderate sleep restriction have not been characterised previously. In the present study, we report no differentially expressed individual genes in the skeletal muscle samples of those with an 8-h sleep opportunity (NS group) or those with a 4-h sleep opportunity (SR group). This result contrasts with a previous study that investigated the skeletal muscle transcriptional response to one night of total sleep deprivation (13). Cedernaes et al. (13) reported that 118 skeletal muscle mRNA transcripts were altered (20 up-regulated and 98 down-regulated), including genes associated with inflammatory processes, protein degradation, mitochondrial function, and glucose metabolism. While there were no differentially expressed individual genes in our study, the gene set composed of the down-regulated genes from Cedernaes et al. (13) (e.g., SD – Down) was also significantly decreased in our SR group; this suggests that our sleep intervention may have had some similar but smaller effects on the transcriptome.

Like Cedernaes et al. (13), we report an increased enrichment of inflammatory and immune-related pathways, and a negative enrichment of pathways associated with mitochondrial regulation (including oxidative phosphorylation-related pathways) in the SR group. The changes in these mitochondrial and inflammatory-related pathways may contribute to reductions in glucose tolerance and mitochondrial function previously reported following sleep loss (7, 11, 13, 15, 16). Of note, changes in glucose tolerance (7, 11), blood pressure and vascular endothelial function (52), inflammatory markers (e.g., IL-6 and TNF-α) (53), and neuro-behavioural assessments (54) significantly differ depending on the dose and extent of sleep loss. Therefore, the differences in the magnitude of the transcriptomic response in this study compared to previous reports may be explained by the use of different sleep loss interventions and the severity of the sleep loss.

The duration of our intervention may also have contributed to differences observed between this study and that of Cedernaes et al. (13). There is evidence to suggest the short-term effects of a period of sleep loss, such as one night of sleep deprivation (11, 13), an extended period of sleep restriction (7), or even a single night of sleep restriction (9), reduces glucose tolerance; however, the longer-term effects may not be as pronounced (55, 56). For example, Robertson et al. (55) reported that insulin sensitivity (determined *via* a hyperinsulinaemic–euglycaemic clamp) was decreased following one week of sleep restriction (habitual sleep reduced by 1.5 h each night), but this effect was no longer evident after two or three weeks of the same intervention. Furthermore, Sweeney et al. (56) assessed glucose tolerance after each night of sleep restriction (4 h TIB for 4 nights) and observed that although glucose tolerance was reduced with SR, this impairment did not occur in a cumulative manner. Similarly, sleep-loss-induced changes in blood pressure and endothelial function are highest after one night of sleep restriction (4 h TIB) and gradually decrease over multiple nights (57), and increased energy intake is reported in the early phases of sleep restriction protocols before returning to baseline levels on subsequent nights (58, 59). These findings suggest an adaptive physiological response to the stress of sleep restriction that occurs over extended periods; this same phenomenon may help to explain the results in this study. In the same way that there appears to be a diminished transcriptional response to the stress of exercise when the same exercise session is repeated (37, 60, 61), the transcriptional response to the stress of sleep restriction may decrease over time. Similar models of this phenomenon, termed allostasis, have been proposed in this field previously (62, 63). How this hypothesis fits with epidemiological evidence of long-term deleterious effects of inadequate sleep requires further investigation.

A curious aspect of our findings was the similar direction of change in enrichment of many GSEA pathways for both the SR and NS groups. Noticeably, there was a significant change in enrichment in 27 of the top 30 pathways for the NS group. While the change in enrichment score was higher in the SR group compared to the NS group in 28 of the 30 pathways (**Supplementary Table 3**), and only the SR group had significant changes in a sleep deprivation gene set (i.e., SD-Down), the similar responses of the NS and SR groups raise the possibility that alterations to these pathways may have been influenced by factors other than sleep restriction. For example, changes in a participant’s environment, diet, and physical activity patterns (although habitual step counts were maintained in our study) have been reported to influence skeletal muscle transcriptomic responses (64, 65). Although habitual step counts were maintained throughout the study, the negative enrichment in a number of mitochondrial-related pathways may reflect changes to regular additional habitual physical activity.

Exercise has been proposed as a potential intervention to mitigate the detrimental metabolic effects associated with inadequate sleep (18). In this study, the GSEA indicated that several pathways associated with mitochondrial regulation had increased their enrichment post-intervention in the SR+EX group, compared to the SR and NS groups. HIIE is well known to induce increases in mitochondrial content and respiratory function (29, 66), which may explain why these pathways were positively enriched in our study. Furthermore, despite the increased enrichment of inflammatory and immune pathways [i.e., Interferon alpha/beta signalling (R-HSA-909733)] in the SR group, this change was not evident in the SR+EX group. It has been proposed that exercise can induce a protective, anti-inflammatory environment (67), which may explain why the increases in these pathways observed in the SR group were not observed in the SR+EX group. Furthermore, there was a significant positive enrichment in the SR+EX group for the SD – Down gene set, suggesting that the transcriptional response of these genes was in the opposite direction to both the observed change in the SR group and previously reported following sleep deprivation. Collectively, these results suggest that the transcriptional response to HIIE during a period of SR prevented the increases in interferon signalling pathway enrichment that were reported with SR alone.

The timing of the collection of muscle samples may influence the interpretation of transcriptional responses within skeletal muscle that have been reported in both sleep loss and exercise studies. In the present study, muscle samples were collected three hours after waking from the final night of sleep restriction (4 h TIB). Considering that the homeostatic drive for sleep increases throughout the day, it is plausible that despite experiencing significant sleep debt throughout the study the transcriptional changes with this type of intervention may occur later in the day or have been diminished following four hours of sleep (68). The timing of muscle sample collection may also explain why no differentially expressed individual genes were observed in the SR+EX group. High-intensity exercise protocols like that used in this study can elicit significant transient, transcriptional changes within skeletal muscle that typically return to resting values within 24 to 48 h (38, 69). Muscle samples in our study were purposely collected 48 h following the final HIIE session to avoid capturing the acute effects of exercise, but this also means it is likely our analysis did not detect skeletal muscle transcriptional changes that occurred in the time immediately post exercise. Indeed, Mahoney et al. (70) assessed global mRNA expression in skeletal muscle *via* cDNA microarray following an exhaustive 75-minute bout of HIIE. It was reported that 181 genes were differentially expressed 3 h post exercise, but this number had reduced to 41 genes by the 48-h time point (which corresponds to the timing of the sample collection following the final HIIE session in the present study). Therefore, the timing of sample collection in this study may not have captured the transient nature of all the transcriptional changes induced by our interventions.

One previous study has investigated the influence of sleep restriction on changes to the transcriptome of peripheral blood cells (71). In this study, participants underwent a sleep restriction protocol of seven nights, with 6 h TIB each night (average sleep duration of 5.7 h). It was reported that 711 gene transcripts were altered following the intervention (267 up-regulated and 444 down-regulated). While the intervention used is of a similar nature to the present study, it is difficult to compare the transcriptional results between different tissues. While Moller-Levet et al. (71) and others (72) have suggested that changes to the blood transcriptome may be reflective of the changes within other tissue, this may not necessarily be the case in skeletal muscle. Recently, a meta-analysis on transcriptomic responses to a single session of exercise in skeletal muscle compared to blood was performed (39). The gene set enrichment analysis (GSEA) found overlap of just one enriched pathway between muscle and blood. Furthermore, in Cedernaes etal. (13), the transcriptomic response of skeletal muscle and adipose tissue to sleep deprivation was compared. There was no overlap between the two tissues for differentially expressed transcripts, highlighting the tissue-specific effects induced by sleep loss. As such, it is not necessarily surprising that the present study’s findings are different to previously reported transcriptional responses from whole blood.

In comparison to other studies investigating the skeletal muscle transcriptional responses to sleep deprivation (13) and the whole-blood transcriptome response to sleep restriction (71), the current study has a smaller sample size and a parallel group design (rather than a cross-over design). Furthermore, the inter-individual coefficient of variation for whole-blood transcripts at baseline and in response to exercise has previously been reported to be approximately 27% (73). Therefore, the smaller sample size of this study, coupled with the heterogeneity of transcripts at baseline and in response to the sleep intervention, would have limited the ability to detect meaningful changes in the samples. Another caveat of the study design is that muscle samples were collected following a standardised (75 g) 2 h OGTT. Considering that insulin has a known influence on the skeletal muscle transcriptome (74) and that participants in the SR group had an altered glycaemic response (15), this may have impacted the results. There is a need for follow-up studies with larger sample sizes (that also contain females and older participants) to confirm these findings and increase their application within the wider community. Future studies may also consider including additional muscle sampling time-points. Samples collected after each night of sleep restriction, immediately prior to sleep, and in the hours following exercise may help to elucidate the transcriptional effects of similar interventions and provide insight into the mechanisms underlying the detrimental health effects of inadequate sleep. Furthermore, to improve our understanding of the effect of exercise on the skeletal muscle transcriptome in the context of sleep restriction, an additional group that performed exercise with normal sleep conditions (i.e., NS+EX) would have been valuable. Meal timing and sleep schedules were tightly controlled in our study, however, under free-living conditions periods of sleep restriction are often accompanied by inconsistent schedules and varied eating habits (75). This should be considered when assessing the generalisability of our findings.

In summary, five nights of sleep restriction (4 h TIB, each night) decreased the enrichment of gene pathways associated with mitochondrial function and increased the enrichment of inflammatory-related pathways. In contrast, performing three sessions of HIIE counteracted the pattern of gene set enrichment observed in the SR group, suggesting that exercise may help mitigate the previously reported detrimental effects associated with inadequate sleep. The alterations to the transcriptome in this study were less apparent than that previously reported following 24 h of sleep deprivation (13), suggesting that periods of repeated moderate sleep restriction may not cause the same magnitude of transcriptional response in skeletal muscle compared to more severe interventions.

## Data Availability Statement

The original contributions presented in the study are publicly available. The transcriptomic data has been deposited in the NCBI and can be found under the BioProject PRJNA807098.

## Ethics Statement

The studies involving human participants were reviewed and approved by Victoria University Human Research Ethics Committee. The patients/participants provided their written informed consent to participate in this study.

## Author Contributions

NS, DB and JB were involved in the conception and design of the work, NS, ML, NP, AG, JK, NC, JB, NJ, WL, XW, HC, DB and JB were involved in the acquisition, analysis or interpretation of the data for the work. NS, ML, NP, AG, NC, JB, NJ, JK, DB and JB were involved in drafting the work and revising it critically for important intellectual content. All authors approved the final version of the manuscript. All authors agree to be accountable for all aspects of the work in ensuring that questions related to the accuracy or integrity of any part of the work are appropriately investigated and resolved. All persons designated as authors qualify for authorship, and all those who qualify for authorship are listed.

## Funding

This publication was supported in part by a Sports Medicine Australia (SMA) Research Foundation Grant and Australian Postgraduate Award PhD Scholarship to NS. In addition, support was provided by Overseas Outstanding Tutor Project (2021-2023), Department of Science and Technology of Guangdong Province to WL, XW and HC.

## Conflict of Interest

The authors declare that the research was conducted in the absence of any commercial or financial relationships that could be construed as a potential conflict of interest.

## Publisher’s Note

All claims expressed in this article are solely those of the authors and do not necessarily represent those of their affiliated organizations, or those of the publisher, the editors and the reviewers. Any product that may be evaluated in this article, or claim that may be made by its manufacturer, is not guaranteed or endorsed by the publisher.
